# Comparing burnout and work-life balance among specialists in internal medicine: the role of inpatient vs. outpatient workplace

**DOI:** 10.1186/s12995-021-00294-3

**Published:** 2021-02-09

**Authors:** Felix S. Hussenoeder, Erik Bodendieck, Franziska Jung, Ines Conrad, Steffi G. Riedel-Heller

**Affiliations:** 1 Institute of Social Medicine, Occupational Health and Public Health, University of Leipzig, Ph.-Rosenthal-Str. 55, 04103 Leipzig, Germany; 2 General Practice, Dresdener Str. 34 a, 04808 Wurzen, Germany

**Keywords:** Burnout, Work-life balance, Physicians, Workplace, Inpatient vs. outpatient, MBI, TKS-WLB

## Abstract

**Background:**

Compared to the general population, physicians are more likely to experience increased burnout and lowered work-life balance. In our article, we want to analyze whether the workplace of a physician is associated with these outcomes.

**Methods:**

In September 2019, physicians from various specialties answered a comprehensive questionnaire. We analyzed a subsample of 183 internists that were working full time, 51.4% were female.

**Results:**

Multivariate analysis showed that internists working in an outpatient setting exhibit significantly higher WLB and more favorable scores on all three burnout dimensions. In the regression analysis, hospital-based physicians exhibited higher exhaustion, cynicism and total burnout score as well as lower WLB.

**Conclusions:**

Physician working at hospitals exhibit less favorable outcomes compared to their colleagues in outpatient settings. This could be a consequence of workplace-specific factors that could be targeted by interventions to improve physician mental health and subsequent patient care.

## Background

Physicians have a higher risk of both increased burnout and lowered work-life balance (WLB) as compared to the general population [1, 2]. Burnout can be a consequence of chronic workplace stress and it is characterized by feelings of energy depletion or exhaustion; increased mental distance from one’s job, or feelings of negativism or cynicism related to one’s job; and a decrease in professional efficacy [3]. Consequently, burnout is often understood as a three-dimensional construct, consisting of exhaustion, cynicism and (reduced) professional efficacy [4], and it is often connected to unfavorable WLB [5–7].

Due to physicians` position in the health system, their working situation and mental health have implications for the population. Research shows that physicians` WLB and burnout are connected to medical errors [8–10]. In addition, physician burnout is associated with reductions in professional work effort, increased risk of patient safety incidents, lower quality of care, and reduced patient satisfaction [11–13]. Han et al. [14] conservatively estimate that approximately 4.6 billion USD related to physician turnover and reduced clinical hours can be attributed to burnout each year in the United States.

The majority of physicians worldwide either work in hospitals or practices, and both work places differ in many ways, e.g., with regard to autonomy, work tasks, or responsibility. In their meta-analysis, Roberts et al. [15] found that outpatient-based physicians reported more exhaustion than their inpatient-based colleagues. In their comparative national study the authors showed that physicians in hospitals were more likely to score low on personal accomplishment, a scale similar to professional efficacy, and that they were also more likely to agree that their work schedule leaves enough time for their personal life and family [16]. The study reflects the situation in the US, the meta-analysis includes research from different nations like Hungary [17], Argentina [18] or Australia [19]. Since national health systems, regulations, and financing fundamentally impact working conditions of physicians, results from one country cannot easily be assumed to be valid in another context. Hence, with this study we want to fill a gap in the literature and analyze in how far physicians` workplaces – hospital vs. practice – are associated with burnout and WLB. Our focus is on internists, since they work in inpatient as well as outpatient settings.

## Methods

### Study design and sample

In September 2019, 25% of physicians working in the Federal State of Saxonia, a region in the Eastern part of Germany, were randomly selected and asked to fill out a questionnaire on work strain, health, and work satisfaction, resulting in a sample of 1412 physicians from different specialties. For the analysis in this article we included a subsample of specialists in internal medicine working full time, either in a hospital or in a practice. After removing 67 participants due to missing values, the sample for analysis contained 183 persons.

### Assessment

We assessed whether physicians worked in a practice or at a hospital. In addition, sociodemographic data including age (in years), gender, marital status, and children living in the household (yes vs. no) were assessed. In Germany, physicians working full time at hospitals with few exceptions have similar work contracts with around 42 h per week and little variation in wages (collective agreement). Physicians in their own practices are freelancers that do not receive a fixed salary. Therefore, we limited our analysis to physicians working full time, but did not include working hours or salary as control variables.

### Burnout

In order to assess burnout, we used the German version of the Maslach Burnout lnventory - General Survey (MBI-GS, [4, 20] which consists of three dimensions (1) exhaustion, (2) cynicism, and (3) professional efficacy. In order to compute an individual burnout score, the professional efficacy scale was inverted, and weighted average scores of all three scales were added following the approach by Kalimo et al. [21]. While in theory burnout scores could range from 0 (= never) to 6 (= every day), in the sample they were between 0.16 and 4.67.

### Work-life balance

Global, subjective WLB was assessed with the German Trierer Kurzskala (TKS-WLB, [22]) consisting of five statements that can be answered on a Likert-scale from 1 (= absolutely not true) to 6 (= absolutely correct).

### Statistical analyses

SPSS Version 25 was used for the statistical analysis. We compared means between internists working at a hospital and those working in a practice using independent t-tests. We used multiple linear regressions to analyze the effects of working place on burnout and WLB controlling for age, gender, marital status, and children living in the household.

## Results

### Descriptive characteristics

Our dataset contained 183 individuals of which 51.4% were female. Table 1 shows the general characteristics of the study population.

**Table 1:** General characteristics of the study population

	Total *(N = 183)*	Hospital *(N = 139)*	Practice *(N = 44)*
Age (Mean)***	40.6 (10.8)	36.8 (8.9)	52.5 (7.1)
Gender (female) n.s.	94 (51.4%)	70 (50.4%)	24 (54.5%)
Marital Status***			
Married	93 (50.8%)	60 (43.2%)	33 (75.0%)
In a relationship	62 (33.9%)	59 (42.4%)	3 (6.8%)
Single	28 (15.3%)	20 (14.4%)	8 (18.2%)
Children in the household („yes“)*	82 (44.8%)	56 (40.3%)	26 (59.1%)

***Note***: *p ≤ 0.05; +**p ≤ 0.01; ***p ≤ 0.001; *n.s.* = not significant (referring to differences between “Hospital” and “Practice”). Continuous variables are given as mean (standard deviation), and *p*-values refer to independent t-tests; categorical variables are displayed as numbers (percentages), and *p*-values refer to Chi ^2^ –tests

### Burnout and WLB: differences between physicians working at hospitals vs. practices

Table 2 shows raw mean differences in burnout and WLB between internists working at a hospital and those working at a practice. Internists working at a practice exhibit a lower burnout score and higher WLB.

**Table 2:** Differences in burnout and work-life balance between specialists in internal medicine working at a hospital vs. a practice (*N* = 183)

	Hospital (***N*** = 139)	Practice (***N*** = 44)	Significance
Burnout			
Exhaustion	2.7 (1.3)	2.0 (1.4)	***P*** **= 0.004**
Cynicism	1.8 (1.3)	1.0 (0.9)	***p*** **≤ 0.001**
Professional efficacy	5.0 (0.8)	5.6 (0.5)	***p*** **≤ 0.001**
Burnout score^a^	1.9 (1.0)	1.2 (0.8)	***p*** **≤ 0.001**
Work-life balance	3.1 (1.1)	4.0 (1.2)	***p*** **≤ 0.001**

The significance of *mean differences was analyzed using independent t-tests*

^a^Computation of the total burnout score according to Kalimo et al. (2003)

***Note*:** *p ≤ 0.05; +**p ≤ 0.01; ***p ≤ 0.001

### Workplace as a predictor of burnout and WLB

Table 3 shows the regression analysis with workplace as a predictor of burnout and WLB. Physicians working in hospitals exhibit significantly lower WLB and higher exhaustion, cynicism, and total burnout score than their colleagues working in practices, but there is no effect of workplace on professional efficacy. Being male and having no children in the household both increased WLB, while professional efficacy is positively associated with age and negatively with “being in a relationship” (as opposed to being “married” or “single”).

**Table 3:** Prediction of burnout and work-life balance by workplace (*N* = 183, unstandardized regression coefficients)

	Exhaustion	Cynicism	Professional efficacy	Burnout score^a^	Work-life balance
Workplace (hospital^b^)	−.65*	−.71*	.16	−.52*	.72**
Age	−.00	−.00	.02*	−.01	.01
Gender (male^b^)	.38	.17	.07	.18	−.38*
Marital status (married^b^)					
In a relationship	.13	.01	−.30*	.14	−.30
Single	−.00	−.11	−.17	.02	−.21
Children in household (none^b^)	.29	−.09	.03	.08	−.46*
Constant	2.45	1.90	4.59	1.97	3.26
R^2^	0.07	0.08	0.15	0.10	0.16

^a^Computation of the total burnout score according to Kalimo et al. (2003)

^b^The category coded as “0” (= reference category) is presented in brackets

***Note***: *p ≤ 0.05; +**p ≤ 0.01; ***p ≤ 0.001

## Discussion

Our multivariate analysis showed that there were significant differences between internists working at a practice and those at a hospital in terms of WLB and all dimensions of burnout. Once we include sociographic control variables for the regression analysis, working at a practice still predicted lower exhaustion, cynicism, and total burnout as well as higher WLB.

While our study shows that inpatient-based physicians exhibit more exhaustion, the opposite is the case in the meta-analysis of Roberts et al. [15]. To some extent this could be explained by the fact that in the meta-analysis studies often refer to specific groups of physicians other than internists, like palliative care [23] or psychiatrists [24]. However, it could also be a result of the specific German situation with recent changes in the health system that were connected to physician dissatisfaction [25]. These changes affected financing, hospital capacities and privatization of hospitals, putting pressure especially on physicians working at hospitals which matches with the results from a recent study that connects working in inpatient settings with an increased wish to leave clinical work [26]. Analogous, these changes could have contributed to the higher cynicism scores in hospital-based physicians in our study which are not reflected in the international meta-analysis [15].

The described differences in exhaustion and cynicism are also reflected in the total burnout score. Following the classification of burnout scores by Kalimo et al. [21], the average score of physicians in outpatient settings (1.2) would be categorized “no burnout” (0–1.49), while the average score of their inpatient colleagues (1.9) would be classified as “some burnout symptoms” (1.50–3.45). In addition, 55.4% of physicians in hospitals would be categorized as having “some burnout symptoms”, and 4.3% would be classified as “burnout” (vs. 36.4%/ 0.0% for physicians working in practices). The fact that physicians working in inpatient settings exhibit higher burnout scores than their outpatient colleagues is supported by similar results from a study of urologists in another German state [27]. The differences could be a consequence of risk factors connected to hospital work itself like high workload [28, 29], limited autonomy [30, 31], occupational stress [32], or a mismatch between own values and those of the management [33], or a consequence of the specific characteristics of hospital physicians which are, for example, younger on average and therefore more prone to experiencing negative consequences from work-life interfering with family [34]. In addition, the results match with recent survey data showing that hospital physicians worked around 6.7 h overtime per week [35]. Future research should address interventions and strategies to mitigate burnout risk specifically at hospitals. For example, these measures could include reduced working schedules, fines for employers that systematically pressure physicians to work overtime, contracts that allow more flexibility for physicians, burnout prevention during studies, and working towards a culture that values physician health.

Doctors working at hospitals were more likely to score low on personal accomplishment/ professional efficacy in the national comparison study by Roberts et al. [16] as well as in our multivariate analysis, but the difference disappeared in the regression analysis once control variables had been added. In our sample, relationship status and even more age were associated with professional efficacy. Therefore, the higher scores of outpatient physicians may to some extent be a consequence of the fact that they were more than 15 years older on average.

WLB was higher for physicians working in an outpatient setting which matches with our results on burnout, but is different from the results of the national comparison study of US internists where recent work-home conflict was similar between groups and hospital-based physicians were more likely to agree that their work leaves enough time for their personal life [16]. The associations between reduced WLB and female gender as well as children in the household were also reflected in other research [17, 36].

### Limitations

While this study has several advantages, e.g., being the first of its kind utilizing a German sample, our research also has certain limitations. First, we did not assess the number of years a physician spent at their workplace which may have an impact on burnout and WLB. Second, we excluded a relatively large number of participants (*N* = 67) due to missing values. Third, future research may benefit from assessing interactions between burnout and WLB with a longitudinal design, and from further differentiating our workplace variable, e.g., with regard to hospital funding or size.

## Conclusions

International research shows that physicians exhibit more burnout and less WLB than the general population [1, 2]. We were interested in how far physicians` workplace is connected to these phenomena in a German setting, and our study suggests that hospital-based physicians are more affected. Hence, targeted healthcare interventions and reforms are needed to improve the situation of physicians working at hospitals in order to maintain the medical infrastructure in the long run and to improve the healthcare system for the benefit of physicians and patients alike.

## Data Availability

The dataset used and analysed during the current study is available from the corresponding author on reasonable request.
